# The Effect of Kinesio Taping Combined with Virtual-Reality-Based Upper Extremity Training on Upper Extremity Function and Self-Esteem in Stroke Patients

**DOI:** 10.3390/healthcare11131813

**Published:** 2023-06-21

**Authors:** Seo-Won Yang, Sung-Ryong Ma, Jong-Bae Choi

**Affiliations:** 1 Department of Occupational Therapy, Sangji University, 83 Sangjidae-gil, Wonju-si 26339, Republic of Korea; yje@hallym.ac.kr; 2 Department of Occupational Therapy, Chosun University, 309 Pilmun-daero, Dong-gu, Gwangju 61452, Republic of Korea; masung79@chosun.ac.kr

**Keywords:** stroke, upper extremity rehabilitation, hand function, virtual reality, Kinesio Taping

## Abstract

(1) Background: The purpose of this study is to investigate the effect of virtual-reality-based hand motion training (VRT) in parallel with the Kinesio Taping (KT) technique on upper extremity function in stroke patients and to present a more effective therapeutic basis for virtual reality training intervention. (2) Methods: First, 43 stroke patients were randomly assigned to two groups: 21 experimental subjects and 22 controls. The experimental group performed Kinesio Taping (KT) on the dorsal part of the hand along with virtual-reality-based hand motion training, and the control group performed only virtual-reality-based hand motion training. The intervention was conducted for a total of 30 sessions over 6 weeks. To evaluate changes in upper extremity function, the Fugl–Meyer Assessment of the Upper Extremity (FMA-UE), the Wolf Motor Function Test (WMFT), and the Motor Activity Log (MAL) (including amount of use (AOU) and quality of movement (QOM)) were evaluated. In addition, the Self-Efficacy Scale (SEF) was evaluated to examine the change in the self-esteem of the study subjects. (3) Results: The experimental group who participated in the virtual reality training in parallel with the KT technique showed statistically significant improvement (** *p* < 0.01) in the FMA-UE, WMFT, and MAL evaluations that investigate changes in upper extremity function. SEF evaluation also showed a statistically significant improvement (** *p* < 0.01). A statistically significant difference between the two groups was observed in the evaluation of FMA-UE, WMFT, MAL-QOM, and SEF (^†^ *p* < 0.05), showing that that combined intervention was more effective at improving upper extremity function than the existing VRT intervention. There was no statistical difference between the two groups in the MAL-AOU item, which is an evaluation of upper extremity function (*p* > 0.05). There was a statistically significant difference between the two groups in the amount of change in upper limb function (^††^ *p* < 0.01). (4) Conclusions: It was confirmed that virtual-reality-based hand motion training performed in parallel with the KT technique had a positive effect on the recovery of upper extremity function of stroke patients. The fact that the KT technique provided stability to the wrist by assisting the wrist extensor muscles appears to have improved the upper extremity function more effectively than VRT alone.

## 1. Introduction

Stroke is a disease in which brain function is impaired because the blood supply is interrupted by blockage or rupture of a blood vessel in the brain due to a thrombus [1]. Stroke is the third leading cause of death in South Korea, and its aftereffects incur significant social costs [2]. Neurological symptoms in patients with stroke include motor, sensory, cognitive, language, and mood disorders, and these symptoms appear complex [3]. Among them, hemiplegia is the most common symptom in patients with stroke, and it causes movement disorders, which limit the range of daily living and social activities patients can participate in [4,5]. Restrictions on daily living and social activities in patients with stroke lead to a decrease in their quality of life and self-esteem. Therefore, to restore the ability to perform daily-living activities in patients with stroke, the upper extremity function should be restored to the hemiplegic side. Through this, basic daily-living activities such as eating, dressing, and hygiene management could be performed independently [6,7]. Currently, various therapeutic approaches are used in clinical practice to restore upper extremity function in patients with stroke, with evidence of their therapeutic effects [8,9]. A review of the literature on therapeutic approaches related to the recovery of upper extremity function in patients with stroke includes constraint-induced movement therapy, modified constraint-induced movement therapy, repetitive task training, bilateral training, electrical stimulation, virtual reality training (VRT), and robotic training [10,11]. Among them, upper extremity function training based on virtual reality has recently been highlighted in the rehabilitation field, for its ability to reorganize the brain through VRT [12]. VRT uses mirror neurons as the neurological background. The scientific principle is presented in Ref. [13]. Mirror neurons are mainly distributed in four places: the lower part of the premotor cortex of the frontal lobe, the lower part of the parietal lobe, the temporal lobe, and the front of the cerebral lobe. Mirror neurons send and receive signals and process information to learn perceived behaviors and movements [14,15]. In addition, various studies have suggested that virtual-reality-based upper extremity training improves upper extremity function in patients with stroke and has a positive effect on the performance of daily-living activities [16,17].

However, the VRT presented in the above literature is mostly shoulder and elbow joint movement training related to large movements, and VRT related to the most elaborate hand movements used in daily-life activities is lacking.

In addition, spasticity among patients with severe hemiplegia, which is the most frequent symptom of stroke, causes difficulty in controlling hand movements in virtual-reality-based fine-motor hand training [18]; thus, a smooth therapeutic approach becomes difficult. Although various interventions have been applied to reduce spasticity in patients with stroke, recent studies have reported the effects of the Kinesio Taping (KT) intervention as well as its advantages in convenience, safety, and economy. Kinesio Taping (KT) has been reported to be effective at (1) reducing inflammation and promoting range of motion through improvement of blood and lymph circulation, (2) relieving pain, and (3) increasing sensory feedback and movement in joints and muscles [19]. Kinesio Taping affects the fascia and stimulates elastic fibers through taping to help control muscle tone along with sensory stimulation during movement [20]. According to recent studies on the relationship between Kinesio Taping and muscle strength improvement, Kinesio Taping does not have a significant effect on muscle strength improvement, but results suggest that it can effect muscle fatigue reduction and support muscle activation [21]. In addition, KT can contribute to the improvement of hand movements by providing stability to wrist extensor muscles [22]. In this study, Kinesio Taping was performed together with virtual reality training to provide sensory feedback for the movement along with wrist extensor support during virtual-reality upper extremity movement training [23]. According to a study by Kim et al., when virtual training combined with Kinesio Taping was performed on patients with ankle instability, it did not affect static balance, but it did have a positive effect on dynamic balance ability. Although it does not help to improve muscle strength, it seems that it can be expected to play a supporting role in muscle activity during movement [24]. Therefore, we tried to find out what kind of changes in upper extremity function and self-esteem of stroke patients might be observed when virtual-reality-based hand motion training was performed together with Kinesio Taping. We intend to provide a complementary treatment basis for training interventions.

## 2. Materials and Methods

### 2.1. Participants

A total of 45 patients with stroke admitted to Rehabilitation Hospital B in Suwon, Gyeonggi-do, South Korea, were eligible for this study. In addition, all study participants signed a consent form for participation in the experiment. The selection criteria were as follows: diagnosed with a stroke within 6 months of the study start date; capable of following instructions, with an MMSE-K (Mini-Mental State Test-Korea version) score of 24 or higher; having a Brunnstrom stage of above 2 for the distal upper limb; capable of performing a slight hand grasp and slight finger extension, and possessing wrist extensor strength below the “poor” grade. The exclusion criteria were as follows: unilateral neglect and hemianopsia, hearing impairment, and intake of drugs related to spasticity. Of the 45 patients, 2 were excluded based on the selection criteria. Finally, a total of 43 patients participated in the study. There were no dropouts during the intervention. The sample size was calculated using the G-Power program 3.1 for the *t*-test of two groups at significance level 0.05, statistical power 0.8, and effect size 0.8 [25].

### 2.2. Procedure

**Figure 1:**
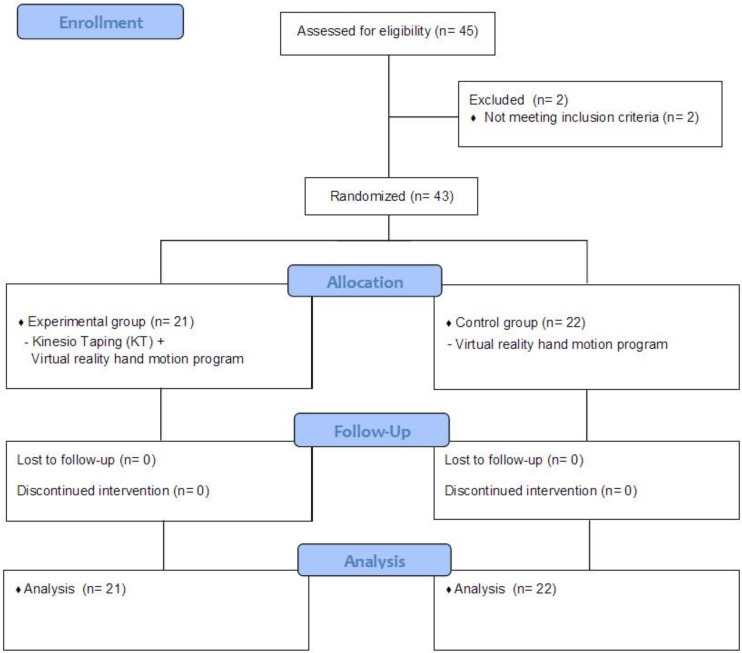
CONSORT diagram of participant recruitment.

This study was conducted from 4 May to 15 November 2020, and a total of 43 patients were divided into the experimental and control groups using a computer randomization method. A computerized (block) randomization scheme was used to randomize participants. The experimental group performed hand movement training based on virtual reality using a motion controller (Leap Motion, LM-010, San Francisco, CA, USA) along with KT (Nitto Kogyo Corporation, Kanihara, Japan), whereas the control group performed only hand movement training based on virtual reality. Both the experimental and control groups were trained for 40 min per session for a total of 30 sessions, 5 times a week for 6 weeks. Figure 1 shows the Consolidated Standards of Reporting Trials (CONSORT) diagram of participant recruitment. 

### 2.3. Intervention

**Figure 2:**
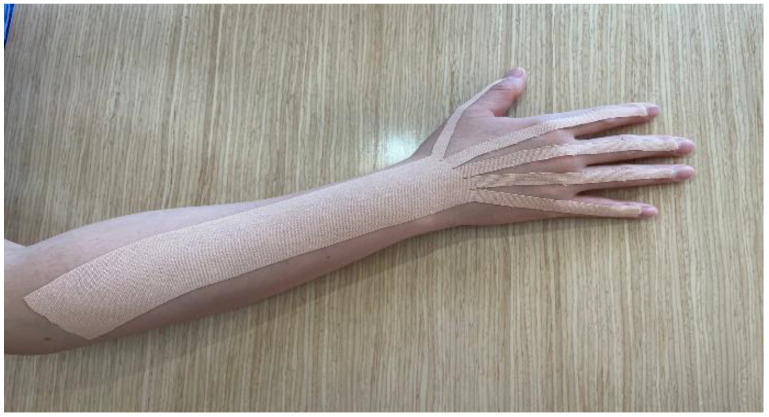
Kinesio Taping applied to the dorsal part on the hemiplegic side.

**Figure 3:**
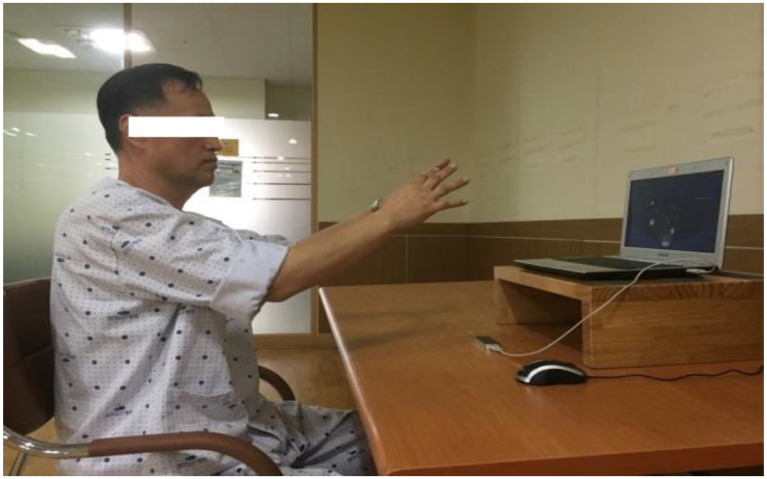
Virtual-reality-based hand motion movement training.

**Figure 4:**
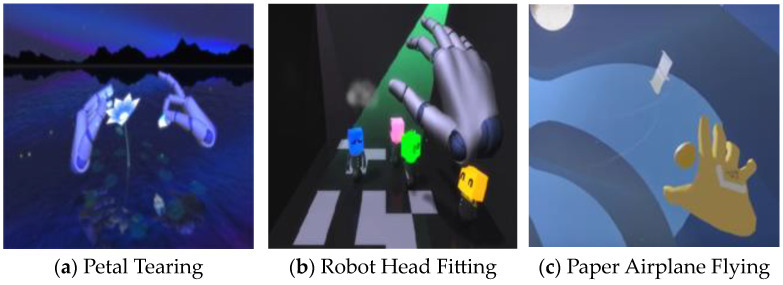

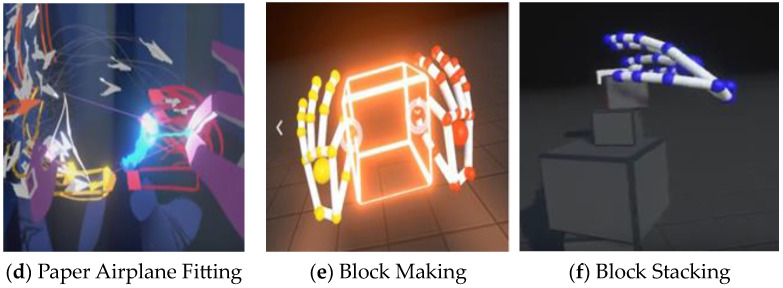
Virtual reality hand motion program.

In the experimental group, Kinesio tape was directly applied by an occupational therapist to the dorsal part of the hemiplegic side, and the tape was attached from the dorsal part of the arm to the distal interphalangeal joint of each finger (Figure 2). The tension applied to the Kinesio tape was set to 50%, and it was set for the purpose of fixing and activating the wrist extensor [22,26]. Then, while sitting on a chair in front of a desk equipped with motion controller equipment, the patients were instructed to perform warm-up hand movements first so that the controller could recognize the movement of the affected hand in a straight-back position. Afterwards, they were asked to perform a training program of their choice (Figure 3). A total of six hand motion movement training programs were offered, based on virtual reality that allows patients to select and perform the program according to the difficulty they want and provide an additional explanation of the execution method according to the difficulty. The training program consisted of (1) petal tearing, (2) robot head fitting, (3) paper airplane flying, (4) paper airplane fitting, (5) block making, and (6) block stacking (Figure 4) [27]. The VRT participants performed warm-up exercises for hand movements, such as fist, scissors, and paper, and individual finger movements for the first 5 min. In the VRT, each participant selected a VRT program for 15 min each in the first and second halves, and a 5 min break was provided between the first and second halves of the program. The control group performed hand motion training based on virtual reality as in the experimental group, but the KT technique was not applied.

### 2.4. Outcome Measures

#### 2.4.1. Fugl–Meyer Assessment for the Upper Extremity (FMA-UE)

The Fugl–Meyer motor function evaluation is a modified Brunnstrom classification for the development of hemiplegia. In this study, only shoulder and elbow (18 items), wrist (5 items), hand (7 items), and upper extremity (3 items) coordination ability were evaluated during the Fugl–Meyer assessment, to evaluate only upper extremity function. Evaluation items were scored 0–2 points depending on the degree of performance: 0 points, not performed; 1 point, partially performed; and 2 points, completely performed. The overall score included all functions of the upper and lower limbs. However, in this study, only the upper extremities were evaluated; thus, the maximum score was 66. The reliability of the inspector for this evaluation tool is 0.96 [28,29].

#### 2.4.2. Wolf Motor Function Test (WMFT)

The WMFT is a tool for evaluating upper extremity function in patients with stroke. The tasks are composed of progressing levels of functional movements of the upper extremities from a low-difficulty task to a high-difficulty task. It is evaluated using performance time, which measures the quantity of movement in the upper limbs, and the functional ability scale, which measures the quality of movement. The function score scale evaluates the quality of movement and consists of a 6-point scale with a lowest score of 0 (not performed) and a highest score of 5 (normal movement). The total score is 75 points, with higher scores indicating better upper extremity function. This evaluation tool has a high reliability of 0.88 [30].

#### 2.4.3. Motor Activity Log (MAL)

The MAL, which was created by Taub in 1993, evaluates the quantitative use and qualitative movement of the affected hand during daily-living activities in patients with stroke, through forced–guided exercise therapy. This evaluation tool consists of 30 items related to daily-life behavior and is divided into Amount of Use (AOU) and Quality of Movement (QOM). The evaluation items include moving, doing housework, communicating, eating, dressing, and manipulating the environment. The tool consists of a 6-point scale, with scores ranging from 0 (not performing at all) to a maximum of 5 (performing as before onset). The evaluation score for each item is calculated by adding the total score and dividing it by the number of evaluation items. The internal consistency of this evaluation tool is a = 0.87 for the MAL-AOU and a = 0.90 for the MAL-QOM. Limits of agreement range from −0.70 to 0.85 for the MAL-AOU and from −0.61 to 0.71 for the MAL-QOM, indicating reproducibility sufficient to detect an individual change of approximately 12–15% of the range of the scale [31].

#### 2.4.4. Self-Efficacy Scale (SEF)

The SEF was developed by Sherer and Maddux (1982), and a modified and supplemented tool for patients with stroke was used in Kim’s study. The general SEF scale refers to the belief in one’s abilities and consists of 14 items on a 10-point scale, ranging from 1 point (not at all confident) to 10 points (completely confident)—the higher the score, the higher the sense of self-efficacy. This evaluation tool has a high reliability of 0.92 [32].

### 2.5. Statistical Analysis

Data collected were statistically processed using SPSS Statistics for Windows, version 18.0 (SPSS Inc., Chicago, IL, USA). For the general characteristics of the study participants, the mean and standard deviation were calculated using descriptive statistics, and homogeneity was verified using the chi-square test. A paired *t*-test was used to examine changes in upper extremity function before and after the intervention in the experimental and control groups, and an independent *t*-test was performed to examine the differences between the two groups. An independent *t*-test was performed to compare the amount of change between the two groups before and after the experiment. The significance level for all statistical data was set at *p* = 0.05. In addition, the effect sizes (Cohen d) of the changed scores between the two groups were calculated. Effect sizes of 0.2, 0.5, and 0.8 represent small, moderate, and large effects, respectively.

## 3. Results

### 3.1. Participants’ Characteristics

The general characteristics of the subjects who participated in the experiment are as follows. A total of 43 patients participated in this study; 21 participants in the experimental group underwent hand motion training based on virtual reality in parallel with KT and 22 participants in the control group received only hand motion training based on virtual reality. No significant difference was found between the two groups according to the participants’ sex, age, stroke type, side of stroke, or time since stroke onset (*p* > 0.05). The general characteristics of the study participants are listed in Table 1.

### 3.2. Comparison of Changes in Upper Extremity Function in Groups before and after Intervention

Upper extremity function changes in the experimental group and control group before and after the intervention are as follows. In the experimental group, the FMA-UE score before and after the intervention improved from 22.00 ± 5.12 to 27.33 ± 5.25, showing a significant difference (** *p* < 0.001). In the control group, the FMA-UE score before and after the intervention improved from 22.41 ± 5.73 to 23.55 ± 6.03, and a significant difference was found (** *p* < 0.001). In the WMFT evaluation, the experimental group’s score improved from 18.48 ± 4.78 to 21.71 ± 5.02, and a significant difference was noted (** *p* < 0.001). The control group also had an improved evaluation score, from 17.41 ± 8.23 to 18.05 ± 5.43 points, showing a significant difference (* *p* < 0.05) (Table 2). Finally, in the MAL-AOU items, the experimental group showed significant improvement (** *p* < 0.001) from 0.91 ± 0.32 before intervention to 2.41 ± 0.51 after intervention, and the control also improved from 1.13 ± 0.34 before intervention to 1.90 ± 0.60 after intervention (* *p* < 0.01). There was no statistical difference between the two groups in the MAL-AOU item (*p* > 0.05). In the MAL-QOM items, the experimental group showed significant improvement (** *p* < 0.001) from 0.94 ± 0.37 before the intervention to 2.09 ± 0.78 after the intervention, and the control group also improved from 1.15 ± 0.30 before the intervention to 2.15 ± 0.56 after the intervention, showing significant differences (** *p* < 0.001) (Table 2). Cohen’s d effect size was 0.74, 0.5, 0.23, and 1.32 for the FMA-UE, WMFT, MAL-AOU, and MAL-QOM, respectively.

### 3.3. Comparison of Changes in Self-Efficacy within Groups before and after Intervention

Changes in self-efficacy in the experimental and control groups before and after the intervention are as follows. In the experimental group, the SEF score improved from 15.33 ± 3.74 points before the intervention to 21.95 ± 4.21 points after the intervention, showing a significant difference (** *p* < 0.001), and the control group also had an SEF score of 15.64 ± 4.5 points before the intervention, which improved to 19.05 ± 4.82 points after the intervention, showing a significant difference (** *p* < 0.001) (Table 2). Cohen’s d effect size was 0.71 for the SEF.

### 3.4. Comparison of Changes in Upper Extremity Function and Self-Efficacy between Groups before and after Intervention

Changes in upper extremity function and self-efficacy between the two groups before and after intervention are as follows. The change in upper extremity function after the intervention between the experimental and control groups showed a significant difference only in the FMA-UE, WMFT, MAL-QOM, and SEF items (^†^ *p* < 0.05), and a significant difference was not noted in the remaining items (*p* > 0.05) (Table 2).

### 3.5. Comparison of Changes in Upper Extremity Function and Self-Efficacy in Groups before and after Intervention

**Figure 5:**
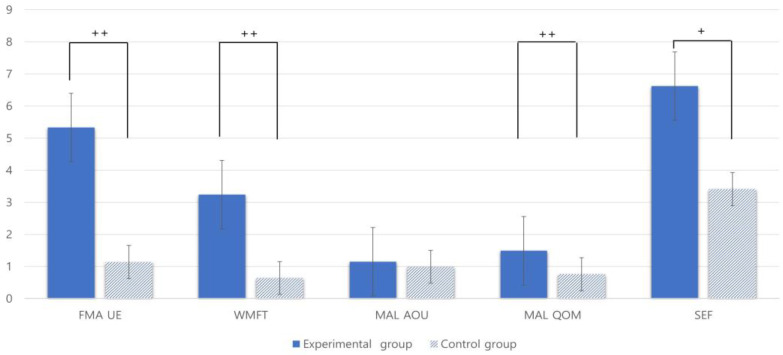
Changes in Both Groups before and after Intervention. The values are mean ± standard deviation, ^†^ *p* < 0.05, ^††^ *p* < 0.001 by independent *t* test.

Changes in upper extremity function and self-efficacy in the experimental and control groups before and after the intervention are as follows. The experimental group showed a significant change in FMA-UE, WMFT, MAL-QOM and evaluation scores, more than the control group (^††^ *p* < 0.01). In the SEF test, the amount of change in the evaluation score was significantly greater in the experimental group than in the control group (^†^ *p* < 0.05) (Table 3, Figure 5).

## 4. Discussion

This study aimed to investigate the effects of virtual-reality-based hand movement training in parallel with KT on upper extremity function and self-efficacy in patients with stroke. In this study, statistically significant improvement was shown not only in the experimental group, in which KT and VRT were performed in parallel, but also in the control group, in which only VRT was performed. In the evaluation results of the experimental group that performed KT and VRT in parallel, the upper extremity function improved overall after the intervention, and functional movements were mainly improved in the wrist and hand as a result of the FMA-UE and WMFT. In the FMA-UE evaluation, scores improved significantly in the movement items on the hand and wrist, and in the WMFT evaluation, the item involved in moving objects using the hands saw a significantly improved score. However, when compared to the minimal clinically important difference (MCID) value of the FMA-UE assessment tool in previous studies, the fact that the statistically significant difference in this study is not always meaningful to patients should be considered in future studies [33]. Following VRT for 18 sessions over 6 weeks in patients with chronic stroke, VRT was effective at improving upper extremity function [34], and VRT using Xbox, which was applied for 6 weeks, revealed consistent results for improving upper extremity function, except for the wrist [35]. However, unlike previous studies, KT was used to provide stability to the wrist and extensor muscles, the distal movement of the hand, and the proximal movement of the upper extremity caused by wrist stiffness and muscle weakness. Functional movement was improved. In addition, the SEF evaluation showed that self-esteem improved compared to before intervention in the experimental group that underwent both KT and VRT. These results were consistent with those in previous studies showing that patients’ pain relief and self-esteem improved following the application of the KT technique in 30 patients with stroke, and [36] a virtual-reality-based game training program improved upper hand function and self-esteem in 42 hospitalized patients [37]. In the comparison between the experimental group and the control group, significant differences were found in the FMA-UE, WMFT, MAL-QOM, and SEF evaluation items. Considering these results, the stability of the wrist and proper extension motion are important factors for more effective restoration of upper extremity function in patients with stroke. There was no statistical difference between the two groups in the MAL-AOU item, which is an upper limb function evaluation, and the reason for this result is that both groups showed improvement in the MAL-AOU item after intervention. Through this, it was found that the frequency of use of the damaged upper limb can be improved only by intervention with virtual reality training. However, there was a difference between the two groups in the MAL-QOM item, indicating that the Kinesio Taping (KT) method, which played an auxiliary role in activating wrist muscles, had a positive effect on qualitatively improving upper limb movement. Moreover, when comparing the change in self-efficacy between the two groups, the experimental group showed a significant change. Restriction of hand function, which appears to be due to stiffness of the wrist and decreased muscle strength, limits participation in many daily-living activities, which appears to affect self-efficacy. The VRT systems used in previous studies have disadvantages in that they require expensive equipment and are subject to spatial limitations in performance. In addition, most of the previous VRT programs only performed training according to the proximal movement of the upper extremity while holding the controller in the hand, so there were many limitations in training for the most sophisticated hand movements used in daily living. Therefore, the KT and VRT systems used in this study are expected to be more effective at improving the distal movement of the upper extremity, that is, the functional movement of the hand.

This study has some limitations. First, the results of the study have limited generalizability due to the small number of participants. Second, it is difficult to ascertain the continuity of the treatment effect on the study participants. We hope that studies with a larger number of participants will be conducted to compensate for these limitations in the future. Prospective studies are needed to investigate the continuity of treatment effects over time. Finally, in the field of occupational therapy, a study is warranted to investigate whether virtual reality intervention for the recovery of upper extremity function in patients with stroke has a positive effect through interaction with other treatments.

## 5. Conclusions

In the results of this study, both VRT training and intervention methods combining VRT and KT were effective at improving the upper hand function of the study subjects, but virtual reality hand motion training combined with Kinesio Taping improved the upper hand function more effectively than the existing virtual reality hand motion training. From the research results, it was found that stability in the wrist is important for recovering the hand function of stroke patients, and the KT used in this study provides stability to the wrist through muscle activation that affects the wrist and finger elongation. Therefore, it seems that functional recovery in the upper extremity was more effective than when only the existing VRT training was conducted. In the future, when applying virtual-reality-based treatment to stroke patients, functional movement of the wrist should be considered. Finally, through this study, it is hoped that various therapeutic grounds for the study of therapeutic interventions based on virtual reality will be presented.

## Figures and Tables

**Table 1:** Characteristics of participants.

Characteristics	Experimental Group (*n* = 21)	Control Group (*n* = 22)	X^2^/t	*p*
Age (year), mean ± SD	59.00 ± 12.66	63.09 ± 9.66	−1.186	0.243
Gender (male/female)	11/10	13/9	0.433	0.667
Type of stroke (Hemorrhage/Infarction)	11/10	15/7	−1.688	0.100
Side of stroke (Right/Left)	12/9	13/9	−0.741	0.463
Time since onset of stroke (months), mean ± SD ^1^	3.81 ± 1.66	3.86 ± 1.28	−0.119	0.906

^1^ SD: standard deviation.

**Table 2:** Comparison of results between experimental group and control group.

	Experimental Group			Control Group			Between Groups *p*-Values
	Before Treatment	After Treatment	*p*-Value	Before Treatment	After Treatment	*p*-Value	
FMA-UE	22.00 (5.12)	27.33 (5.25)	<0.000 **	22.41 (5.73)	23.55 (6.03)	<0.000 **	0.034 ^†^
WMFT	18.48 (4.78)	21.71 (5.02)	<0.000 **	17.41 (8.23)	18.05 (5.43)	<0.036 *	0.027 ^†^
MAL-AOU	0.94 (0.37)	2.09 (0.78)	<0.000 **	1.15 (0.30)	2.15 (0.56)	<0.000 **	0.782
MAL-QOM	0.91 (0.32)	2.41 (0.51)	<0.000 **	1.13 (0.34)	1.90 (0.60)	<0.000 **	0.015 ^†^
SEF	15.33 (3.74)	21.95 (4.21)	<0.000 **	15.64 (4.53)	19.05 (4.82)	<0.000 **	0.041 ^†^

FMA-UE: Fugl–Meyer Assessment for the Upper Extremity; WMFT: Wolf Motor Function Test; MAL-AOU: Motor Activity Log Amount of Use; MAL-QOM: Motor Activity Log Quality of Movement; SEF: Self-Efficacy Scale. The values are mean ± standard deviation, * *p* < 0.05, ** *p* < 0.001 by Paired *t* test, ^†^ *p* < 0.05 by independent *t* test.

**Table 3:** Changes in both groups before and after Intervention.

	Experimental Group	Control Group	*p*-Value
FMA-UE	5.33 (4.32)	1.14 (1.16)	<0.000 ^††^
WMFT	3.24 (2.82)	0.64 (1.32)	<0.001 ^††^
MAL-AOU	1.15 (0.65)	0.99 (0.59)	<0.417
MAL-QOM	1.49 (0.48)	0.76 (0.73)	<0.000 ^††^
SEF	6.62 (4.85)	3.41 (2.88)	<0.011 ^†^

FMA-UE: Fugl–Meyer Assessment for the Upper Extremity; WMFT: Wolf Motor Function Test; MAL-AOU: Motor Activity Log Amount of Use; MAL-QOM: Motor Activity Log Quality of Movement; SEF: Self-Efficacy Scale. The values are mean ± standard deviation, ^†^ *p* < 0.05, ^††^ *p* < 0.001 by independent *t* test.

## Data Availability

The datasets generated during the current study are available from the corresponding author upon reasonable request.
